# Supplementary material for: Fetal demise and failed antibody therapy during Zika virus infection of pregnant macaques
Source: Nat Commun. 2018 Apr 24;9:1624. doi: 10.1038/s41467-018-04056-4 (PMC5915455; doi:10.1038/s41467-018-04056-4)
Supplement: Supplementary file 1 — Supplementary Information [file 41467_2018_4056_MOESM1_ESM.pdf]

Supplementary information

Fetal demise and failed antibody therapy during Zika virus infection of pregnant macaques

Magnani DM et al.

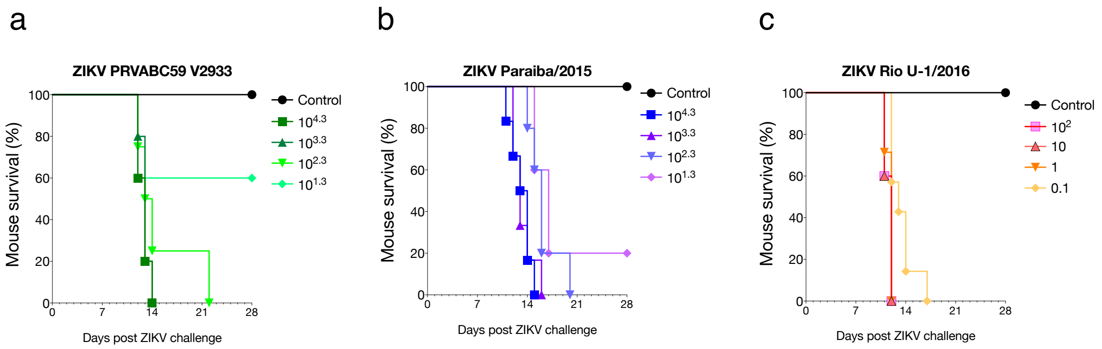

Supplementary Fig. 1. ZIKV Rio U-1/2016 is pathogenic for AG129 mice. Kaplan-Meier survival curves after challenge with different doses (PFU) of ZIKV isolates PRVABC59 (a); Paraiba/2015 (b), or Rio U-1/2016 (c). Male and female mice (groups of 5-6 animals) were inoculated with the doses (PFU) of three different ZIKV isolates and survival was followed for 28 days.

Supplementary Table 1. Study animals

Group	Animal ID	Gestational day at day of ZIKV inoculation	Age at time of ZIKV inoculation	Weight (kg) at time of ZIKV inoculation	Infant ID (fetal outcome)
1st Trimester	M01	44	14.63	6.70	F01 (deceased)
	M02	49	4.76	4.95	F02 (euthanized)
	M03	49	3.77	5.91	F03 (deceased)
2nd Trimester	M04	64	5.92	3.75	F04 (euthanized)
	M05	100	9.60	9.03	F05 (euthanized)
	M06	105	9.77	8.08	F06 (alive)
	M07	107	13.75	8.11	F07 (alive)
3rd Trimester	M08	112	8.28	8.41	F08 (euthanized)
	M09	120	6.31	8.33	F09 (alive)*
	M10	122	9.25	7.19	F10 (alive)*
	M11	124	9.34	10.18	F11 (unresponsive)
Therapy experiment	M12	106	14.06	6.84	F12 (euthanized)
	M13	125	8.97	6.61	F13 (euthanized)
	M14	134	5.97	8.90	F14 (euthanized)
	M15 (untreated)	139	6.82	9.07	F15 (euthanized)

*These two animals were born alive and kept alive for 6 months.

Supplementary Table 2. Summary of ZIKV sequencing

Sample info					Sequencing stats		Variant site (nt, frequency, coverage depth)[b]									
Sample ID	Time (dpi)	Type	ZIKV GE/ul RNA	CDS cover age	ZIKV reads	Ave. cov. depth	637	3053	4740	5548	5592	6472	6499	7505	8888	8963
RIO U-1/2016	0	stock	UD	100%	1,007,502	18,763	**T**	**T**	**G**	**C**	**C**	**C (T 2%, 19316)**	**C**	**C**	**T**	**C**
M12	3	serum	4,635	100%	306,288	5,327	--	--	--	--	--	--	--	--	--	--
M12	5	serum	~1	55.3%	441,201	3,951	--	?	--	?	--	?	?	--	?	?
M12	7	serum	5	91.4%	116,939	1,779	--	--	A (26%, 10081)	--	--	?	?	--	--	--
M12	10	serum	7	82.8%	429,089	6,945	A (35%, 18830)	--	--	--	--	--	?	--	--	--
M15	3	serum	6,582	98.2%	68,645	1,186	--	--	--	--	--	--	T (40%, 241)	--	--	--
M15	7	serum	13	91.9%	325,938	5,771	--	--	--	--	--	?	?	T (41%, 27176)	--	--
M15	10	serum	~1	48.2%	331,494	4,143	?	--	?	?	--	?	?	T (100%, 964)	--	--
M01	3	serum	43	93.3%	1,263,658	21,839	--	--	--	--	--	--	--	--	--	--
M01	3	serum	43	97.9%	536,471	8,997	--	--	--	--	--	?	?	--	--	--
M05	3	serum	2	83.7%	477,124	8,350	--	--	?	--	--	?	?	--	--	?
M05	20	AF	59	89.4%	91,413	1,563	--	C (94%, 668)	--	--	--	?	?	--	--	--
M05	41	AF	36	83.1%	362,283	6,377	--	C (100%, 2789)	--	A (26%, 311)	T (52%, 337)	?	?	--	--	T (100%, 2300)
M09	3	serum	3,203	99.6%	713,830	10,373	--	--	--	--	--	--	--	--	--	--
M09	28	AF	3,489	99.1%	303,059	5,234	--	--	--	--	--	--	--	--	C (55%, 822)	--
M04	3	serum	87	98.5%	934,007	13,311	--	--	--	--	--	--		--	--	--
M04	10	AF	2,401	98.7%	73,412	1,276	--	--	--	--	--	?	?	--	--	--
M07	3	serum	471	97.7%	217,375	3,869	--	--	--	--	--	?	?	--	--	--
M11	3	serum	330	97.9%	839,985	11,333	--	--	--	--	--	?	?	--	--	--
M06	3	serum	2,273	99.2%	383,218	6,775	--	--	--	--	--	T (40%. 1333)	--	--	?	--
M10	3	serum	625	99.4%	687,650	9,759	--	--	--	--	--	--	--	--	--	--
M08	3	serum	482	97.7%	742,999	10,292	--	--	--	--	--	?	?	--	--	--
M13	3	serum	840	98.2%	290,580	5,133	--	--	--	--	--	?	?	--	--	--
M14	3	serum	141	97.9%	391,076	6,905	--	--	--	--	--	?	?	--	--	--
M02	3	serum	1,734	99.1%	280,327	4,988	--	--	--	--	--	?	?	--	--	--
M03	3	serum	136	97.9%	609,080	10,189	--	--	--	--	--	?	?	--	--	--
Male control	3	serum	~500	100%	1,373,000	23,722	--	--	--	--	--	--	--	--	--	--
					Amino acid substitution		prM-M$_{55}$K	silent	NS3-E$_{43}$K	NS3-A$_{312}$D	NS3-P$_{327}$S	NS4A-A$_{3}$V	NS4A-T$_{12}$I	silent	silent	silent

[a]Percent coverage of coding sequence (CDS) at a depth of >25 nt
[b]Called intrahost variants must be present at >25% frequency, >100x coverage, on both forward and reverse reads, and not found in other lab stock viruses. Nucleotides sequenced from the stock virus at these sites are >99.9% in favor of the consensus, unless otherwise specified.
--, no change from ZIKV RIO U-1/2016
?, not sufficient coverage (<100x) at this position to confidently call intrahost variants
